# Water and sewage systems, socio-demographics, and duration of residence associated with endemic intestinal infectious diseases: A cohort study

**DOI:** 10.1186/1471-2458-10-767

**Published:** 2010-12-16

**Authors:** Kay Teschke, Neil Bellack, Hui Shen, Jim Atwater, Rong Chu, Mieke Koehoorn, Ying C MacNab, Hans Schreier, Judith L Isaac-Renton

**Affiliations:** 1 School of Population and Public Health, University of British Columbia, Vancouver, Canada; 2 Department of Civil Engineering, University of British Columbia, Vancouver, Canada; 3 Institute for Resources, Environment and Sustainability, University of British Columbia, Vancouver, Canada; 4 Department of Pathology and Laboratory Medicine, University of British Columbia and BC Centre for Disease Control, Vancouver, Canada

## Abstract

**Background:**

Studies of water-related gastrointestinal infections are usually directed at outbreaks. Few have examined endemic illness or compared rates across different water supply and sewage disposal systems. We conducted a cohort study of physician visits and hospitalizations for endemic intestinal infectious diseases in a mixed rural and urban community near Vancouver, Canada, with varied and well-characterized water and sewage systems.

**Methods:**

Cohort members and their disease events were defined via universal health insurance data from 1995 through 2003. Environmental data were derived from municipal, provincial, and federal government sources. Logistic regression was used to examine associations between disease events and water and sewage systems, socio-demographic characteristics, and temporal factors.

**Results:**

The cohort included 126,499 individuals and approximately 190,000,000 person-days. Crude incidence rates were 1,353 physician visits and 33.8 hospitalizations for intestinal infectious diseases per 100,000 person-years. Water supply chlorination was associated with reduced physician visit incidence (OR: 0.92, 95% CI 0.85-1.0). Two water systems with the highest proportions of surface water had increased incidence (ORs: 1.57, 95% CI 1.39-1.78; and 1.45, 95% CI 1.28-1.64). Private well water and well depth were not associated with increased risk, likely because of residents' awareness of and attention to water quality. There was increased crude incidence with increasing precipitation in the population served by surface water supplies, but this trend did not remain with adjustment for other variables. Municipal sewer systems were associated with increased risk (OR: 1.26, 95% CI 1.14-1.38). Most socio-demographic variables had predicted associations with risk: higher rates in females, in the very young and the elderly, and in residents of low income areas. Increased duration of area residence was associated with reduced risk (OR, duration ≥ 6 years: 0.69, 95% CI 0.60-0.80 *vs*. < 1 year: 1.16, 95% CI 1.03-1.30).

**Conclusions:**

This large cohort study, with objective data on exposures and outcomes, demonstrated associations between endemic infectious intestinal diseases and factors related to water supply, sewage disposal, socio-demographics, and duration of residency. The results did not always follow prior expectations based on studies examining outbreaks and single systems, and underscore the importance of studying factors associated with endemic disease across water and sewage system types.

## Background

Most studies of water and enteric infections have focused on disease outbreaks in single water systems [1]. High profile examples in North America include the 1993 Milwaukee *Cryptosporidium *outbreak where approximately 100 people died and a further 400,000 became ill [2], and the 2000 Walkerton *Escherichia coli *O157 and *Campylobacter *outbreak that caused 7 known deaths and about 2,300 illnesses [3-5].

Few studies have examined non-epidemic illness. Canadian surveillance data suggest that potentially waterborne intestinal infectious diseases account for about 20% of reported communicable disease cases [6,7]. A number of investigations have examined factors influencing endemic intestinal disease within water systems, including turbidity, chlorination, residence time, and system maintenance [8-14]. Studies of endemic disease across multiple water or sewage systems are very rare [15-17].

We investigated the association between the incidence of intestinal infectious diseases and environmental factors potentially contributing to drinking water quality in the Township of Langley in the Metro Vancouver area of British Columbia (BC), Canada. The Township was selected for study, not because of concerns about endemic disease in the area, but because it encompasses rural and urban areas with varied and well-characterized water supply and sewage disposal systems. In addition, individual-level data were available about physician visits and hospitalizations, and about socio-demographic characteristics of the population that might also be associated with disease events.

## Methods

The study methods were reviewed and approved by the University of British Columbia Behavioural Research Ethics Board and by the British Columbia Ministry of Health Services data steward.

### Cohort identification

The cohort was identified by Population Data BC [18]. Its data holdings include the Client Registry of the provincial Medical Services Plan (universal health insurance, estimated to enumerate over 95% of the population), all hospital discharge records, and billing records for visits to physicians. The cohort included all individuals who lived in the Township for at least 6 months between January 1, 1995 and December 31, 2003.

### Disease events

Two types of disease events were identified by linking cohort members to administrative records: a) *Physician visit*, a record in the Medical Services Plan Billings File with a 3-digit International Classification of Diseases, 9^th ^Revision (ICD-9)[19] "diagnostic code" listed in Table 1; and b) *Hospitalization*, a record in the Hospital Discharge Records File with a "most responsible diagnosis" or "primary diagnosis" indicating a 3- to 5-digit ICD-9 code in the range listed in Table 1.

**Table 1:** Number of subjects with physician visits and hospitalizations for intestinal infectious diseases, 1995 to 2003 inclusive, in a mixed rural-urban community in Metro Vancouver.

		Number of subjects with a	
ICD-9 Code*	Disease description	physician visit	hospitalization
003	Other Salmonella infections	65	0
004	Shigellosis	20	0
007	Other protozoal intestinal diseases (includes balantidiasis, giardiasis, coccidiosis, intestinal trichomoniasis, cryptosporidiosis, cyclosporiasis)	21	0
008	Intestinal infections due to other organisms	721	^†^180
009	Ill-defined intestinal infections	6190	0
	Total all diseases above	7017	180

* International Classification of Diseases, 9^th ^Revision [19]

^† ^15 were identified as having either 008.0 *E. coli *or 008.5 Unspecified bacterial enteritis; the remaining 165 were identified as 008.8 Other organisms, not elsewhere classified

The ICD-9 codes were selected in consultations between the study medical microbiologist (JLI-R) and Dr. Robert Fisk of the BC Ministry of Health Services [personal communication, September 2003] to include intestinal infectious diseases with potential to be waterborne and, where possible within the constraints of disease coding, to exclude diseases known *not *to be associated with waterborne disease transmission *in Canada*, such as cholera, typhoid fever, and amoebiasis, and gastrointestinal illnesses known to be predominantly food-borne (e.g., staphylococcal food poisoning, botulism, enteritis necroticans).

### Socio-demographic and temporal variables

Data on *sex *and *birth date *were abstracted from the Client Registry. *Season *was defined as spring (March 1 to May 31), summer (June 1 to August 31), fall (September 1 to November 30) and winter (December 1 to February 29) to correspond more closely to local weather, employment, and school attendance patterns than classical seasonal definitions, and thus to control for seasonal patterns of interpersonal contact that might influence person-to-person transmission (e.g., indoor living, school year). *Duration of residence in the Township *was categorized as < 1 year, 1 - < 2 years, 2 - < 3 years, 3 - < 6 years, ≥ 6 years, and unknown. *Household income quintile of the neighborhood *was derived from Statistics Canada 2001 census data available for each dissemination area (one or more neighboring blocks with a population of 400 - 600 persons), and assigned via residential postal code. *Population density *was calculated as the average number of persons per hectare per year at each property. *Distance to nearest hospital *was calculated from the center of each property to the nearest of two local hospitals, and classified as < 1 km, 1 - < 3 km, and 3 - < 8 km.

### Environmental variables potentially contributing to drinking water quality

Environmental variables were linked geographically via spatial link functions in ArcGIS 9 (ESRI, Redlands, CA) using latitude and longitude coordinates, unique property identifiers, or street addresses, then linked to subjects via street addresses at the Ministry of Health Services.

#### Water system

All addresses supplied by municipal water systems were identified via Township tax records of water connection, further specified as one of 7 categories by location. This allowed distinctions between municipal systems served by wells only (further categorized by the numbers of homes served as small, with < 100 connections, or large systems), and those with mixed water systems supplied from both Township wells and protected surface water reservoirs in the North Shore Mountains (further categorized by relative proportions of surface water in the mix). The municipal wells and distribution systems are managed and tested by the Township, and the reservoirs are managed and tested by the Greater Vancouver Water District. Addresses with water supplied by community systems were identified via Fraser Health Authority records. These systems are managed privately by each community, but tested by health authority officials. All remaining addresses were designated as having private systems (served by on-property wells, with no public health monitoring).

#### Drinking water disinfection

Dates of implementation of chlorination systems were used to indicate presence or absence of chlorine disinfection. Data were provided by the Township for the municipal systems and by Fraser Health Authority for the community systems. All private water supplies were designated as having no disinfection.

#### Sewage disposal

All residential addresses connected to the municipal sewage system were identified via Township tax records of sanitary sewer connection. All other addresses were designated as having private on-property sewage disposal.

#### Land use

Property land uses were classified as agricultural or residential, via BC Assessment records.

#### Well depth

Data were available for a subset of wells from a database based on voluntary reporting by well drillers to the BC Ministry of Environment.

#### Precipitation

Daily precipitation data were downloaded from the Environment Canada National Climate Data for a station in the Coquitlam watershed in the North Shore Mountains, and assigned to properties receiving surface water from the mountain reservoirs.

### Survey of Township households

To gather descriptive data not available from administrative sources, from fall 2006 to summer 2007, we conducted a survey of a random sample (N = 1000) of households with Township postal codes, selected from the CanPages electronic directory. A self-administered questionnaire was mailed, followed by one telephone and two mailed reminders. It included questions about type of water supply, type of sewage disposal, use of tap water for cooking and drinking, filtration of tap water, and for appropriate subsets, questions about well depth and testing of water quality. Agreement between the survey and administrative data on water system type (municipal, community, or private) and sewage system type (municipal or private) was examined by calculating unweighted kappa statistics.

### Data analysis

All statistical analyses were done using SAS 9.1 (SAS Institute, Cary, NC). The unit of analysis was person-day, as most variables were time-varying and cohort membership was dynamic, with individuals moving within and in and out of the Township during the study period.

Initial examinations of the data included identification of intestinal infectious disease events, plots of their occurrence over the study period to check for evidence of epidemics, and examination of multiple event records per subject to ascertain the time periods between records.

Two sets of analyses were conducted, one for physician visits, the other for hospitalizations. Only the first physician visit and first hospitalization for each subject were included in analyses, to exclude the possibility that a subsequent event might be related to the first. Person-days after the first event were excluded. Disease events and person-days within the first 2 months after entry to the cohort (i.e., January 1 1995 for most subjects or the date of moving to the Township if after that date) were also excluded, to allow an incubation period sufficient for any associations to be plausibly related to exposures in the Township.

Person-time and crude disease incidence rates with 95% confidence intervals (CI) were calculated for each category of each independent variable. The statistical estimation and inference reported here are based on fitting the following unconditional logistic regression model, using the full dataset and the PROC LOGISTIC procedure.

(1) $$\log it(P_{it})=\beta_{0}+\sum_{j=1}^{M}\beta_{j}X_{jit}$$

where

$\log it(P_{it})=\log\frac{P_{it}}{1-P_{it}}$, and *P_it _*= the probability of a physician visit (or hospitalization) for an intestinal infectious disease for subject i on day t, and *X_jit _*= value for subject i on day t for each of M variables. (Primary models for *a priori *hypotheses included the variables sex, age, season, duration of residence, household income quintile, drinking water disinfection, water system, sewage disposal, and land use; *post-hoc *analyses added distance to nearest hospital and population density to the primary models).

Log odds ratios and their standard errors were estimated using maximum likelihood methods, and 95% confidence limits were based on the asymptotic normality of parameter estimators. Calendar year was also included in all models, because including only first events meant that they were weighted somewhat more heavily to the initial years. This variable was categorized as 1995-6, 1997-8, 1999-2001, and 2002-3, grouping years with nearly identical crude event rates.

For certain environmental variables apropos only to subsets of the data, secondary analyses were conducted. These analyses were restricted to physician visits only, because there were so few hospitalizations, and included all variables in the primary analyses. In one model, the analysis was restricted to those living at properties whose well depth was known, and well depth was included as an additional variable. In another set of models, analyses were restricted to those living at properties receiving municipal water from surface water reservoirs in the North Shore Mountains, and precipitation was included as an additional variable. Precipitation was calculated as a continuous variable then categorized into 6 categories for modelling (0, 1 to < 10, 10 to < 25, 25 to < 100, 100 to < 250, and ≥ 250 mm). It was calculated in several ways (accumulated millimetres of rain over one- and two-week periods, with no lag and 2-, 5- and 10-day lags), each offered in a separate model.

A number of alternative analysis methods, including Cox proportional hazards and Poisson regression models (classical cohort analysis methods), as well as mixed effects logistic regression and logistic regression with generalized estimating equations (to account for repeated measures on subjects), were considered in the initial stages of data analysis. Due to computational intensity, most of the alternative models were fit on a 10% (1% for the mixed effects model) subsample of the original data. The resulting risk estimates and inferential results remained relatively unchanged, with the exception of some categories in the Poisson regression (likely due to rare events and small person-day counts for these categories in the data subsamples). Of particular note is that random intercept logistic regression, which allows for over-dispersed and correlated outcomes arising from repeated measures and clustered design, was fit on a 1% subsample of the data using PROC GLIMMIX. The estimated variance of the random effects was near 0, which indicates that the mixed effects logistic regression degenerated to regular logistic regression with no random effects. We were unable to run the mixed effects model on the whole data or on a 10% subsample, due to insufficient memory. The GEE logistic regression, which accounts for within-subject correlation in repeated measurement designs, was fit using PROC GENMOD for the whole physician visit dataset, assuming exchangeable within-subject correlation. The estimated effects (the point and interval estimates of the beta coefficients) were nearly identical to those under a comparable unconditional logistic regression model fit using PROC LOGISTIC; the estimated exchangeable working correlation was close to 0.

Note that in this cohort analysis, the follow-up on each individual ended at first observation of a disease event or over the available follow-up years for no event ever observed; this may be the reason for observing small and negligible within-subject correlation in the mixed effects and GEE analyses. The computing time for GEE using PROC GENMOD on the whole physician visit data was about 15 days, while the computing time using PROC LOGISTIC was about 2 days. Therefore, the final models were developed using PROC LOGISTIC, which was computationally most efficient among all methods considered.

## Results

The average population of the Township during the study period was 90,273. The study cohort included 126,499 individuals who resided in the Township for at least 6 months between 1995 and 2003. The analysis dataset for physician visits included 7,017 cases with an intestinal infectious disease and 189,234,765 person-days. The analysis dataset for hospitalizations included 180 cases and 194,552,383 person-days. Person-days differed because the number of days removed after subjects' first events differed. Almost all events had general diagnostic codes not specific to a single organism (Table 1). Plots of disease events by day and week over the study period showed no temporal patterns indicative of outbreaks.

**Figure 1:**
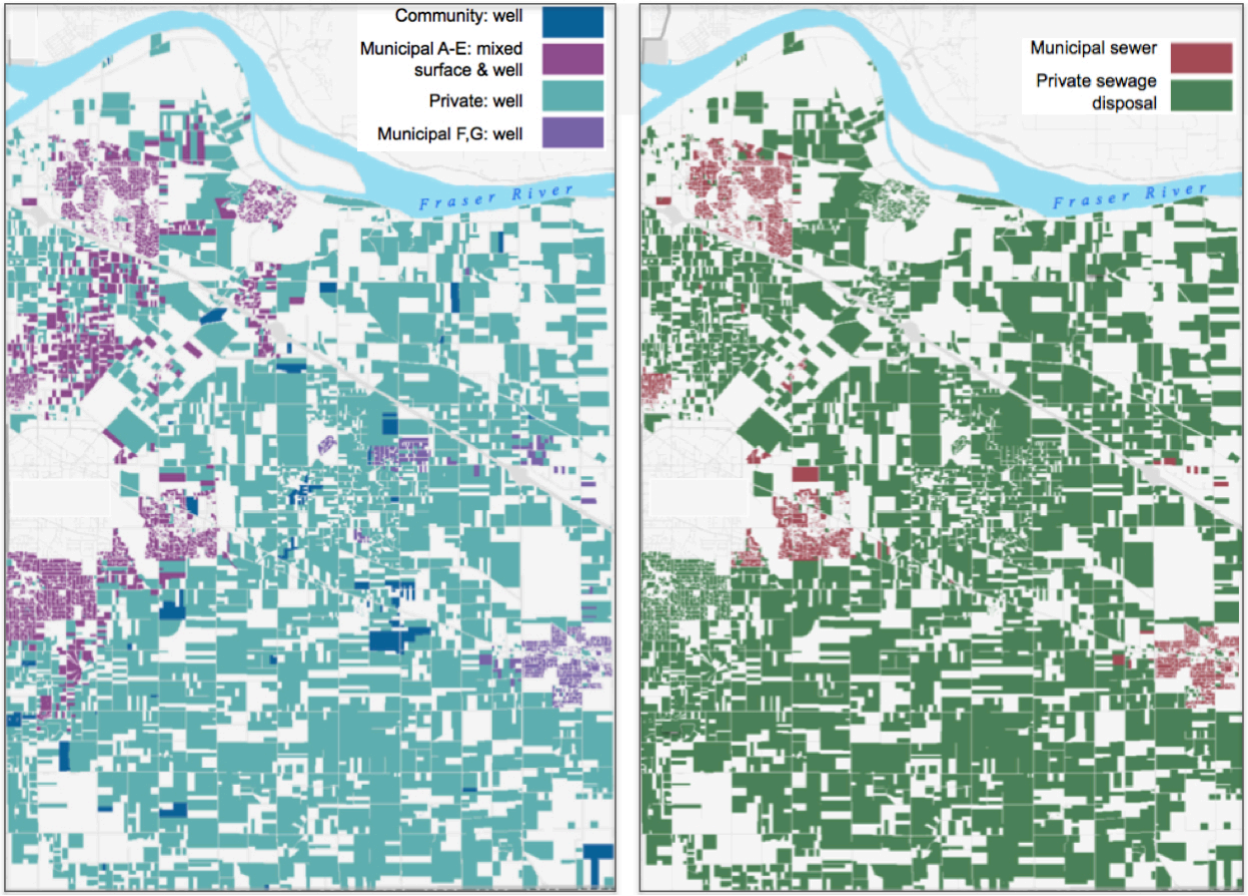
**Maps showing geographic distributions of the water and sewage systems**. Data for parcels in a mixed rural-urban community in the Metro Vancouver area of Canada, on July 1 1999, the mid-point of the study period (of a total of 29,458 parcels). Uncolored areas are parcels not included in the study, either because they were outside the Township or did not house a study subject on that date (e.g., industrial, commercial or park land, or vacant property).

The Township database included 29,458 unique parcels of land (Figure 1). Of the parcels, 30.9% were recorded as private water users only, 66.3% were recorded as municipal water users only, though 69.1% were connected to the municipal water supply at some point during the study period. At the beginning of the study period, no parcels received chlorinated water while over two-thirds (20,387) did by the end of the study, including municipal and community water supplies with surface and well water sources. About half the parcels (49.4%) had private septic systems only, 48.9% were connected to the municipal sewer system only, and 50.6% were connected to the municipal sewer system sometime during the study period. There was a slight decrease in the number of parcels with an agricultural use code from the beginning of the study (8.1%) to the end (7.0%).

Table 2 lists the proportion of person-time for each category of each variable. These differ from the proportions of parcels outlined above, because the numbers of people living at each parcel were not equally distributed by category.

**Table 2:** Proportions of person-time and crude incidence rates of intestinal infectious diseases, 1995 to 2003 inclusive, stratified by categories of each socio-demographic, temporal, and environmental variable.

Variable & Category	Proportion of person-time in each category	Crude rate of physician visits^a ^ per 100,000 person-years [95% CI]	Crude rate of hospitalizations^b ^ per 100,000 person-years [95% CI]
Sex			
Female	50.4%	1,398 [1351, 1351]	35.8 [28.6, 43.0]
Male	49.6%	1,310 [1265, 1265]	31.8 [24.9, 38.5]
Age			
< 1	0.3%	7,848 [6484, 6484]	119 [0, 284]
1 - 4	5.0%	6,607 [6286, 6286]	315 [ 249, 381]
5 - 9	7.4%	2,164 [2014, 2014]	55.8 [33.0, 78.5]
10 - 19	16.2%	902 [838, 838]	21.9 [12.0, 31.6]
20 - 29	11.5%	1,548 [1448, 1448]	11.3 [3.0, 19.9]
30 - 39	15.2%	1,190 [1114, 1114]	6.2 [0.8, 11.6]
40 - 49	17.5%	770 [713, 713]	8.8 [2.7, 14.6]
50 - 59	12.2%	653 [591, 591]	7.7 [1.0, 14.7]
60 - 69	7.2%	756 [667, 667]	24.1 [8.3, 39.8]
≥ 70	7.5%	1,095 [991, 991]	38.3 [19.0, 57.8]
Season			
Spring	25.5%	1,566 [1496, 1496]	56.2 [43.5, 68.7]
Summer	25.6%	1,288 [1229, 1229]	21.2 [13.5, 29.0]
Fall	25.2%	1,128 [1070, 1070]	19.3 [11.9, 26.7]
Winter	23.7%	1,438 [1369, 1369]	38.7 [27.9, 49.7]
Duration of Residence in the Township			
< 1 year	15.0%	2,325 [2219, 2219]	65.7 [47.8, 83.5]
1 - 2 years	17.2%	1,745 [1657, 1657]	46.0 [32.2, 60.0]
2 - 3 years	15.3%	1,226 [1150, 1150]	33.2 [20.7, 45.7]
3 - 6 years	30.8%	923 [876, 876]	21.9 [14.6, 28.9]
≥ 6 years	15.5%	712 [652, 652]	13.1 [5.4, 21.0]
Unknown	6.2%	1,938 [1786, 1786]	37.2 [16.1, 58.0]
Household income quintile of the neighborhood			
Low	4.7%	1,482 [1329, 1329]	63.5 [32.5, 94.8]
Medium Low	10.1%	1,559 [1452, 1452]	40.9 [23.7, 57.7]
Medium	24.1%	1,256 [1194, 1194]	33.6 [23.6, 43.7]
Medium High	43.1%	1,391[1340, 1340]	29.6 [22.5, 36.6]
High	18.0%	1,252 [1178, 1178]	32.1 [20.9, 43.6]
Drinking Water Disinfection			
Chlorination	38.7%	1,343 [1293, 1293]	27.7 [20.7, 35.1]
None	61.3%	1,358 [1319, 1319]	37.6 [30.9, 44.2]
Water System or Subsystem			
Municipal A*: mixed surface (66-96%) & well	27.2%	1,905 [1832, 1832]	28.8 [20.0, 37.3]
Municipal B*: mixed surface (66-96%) & well	8.6%	1,741 [1619, 1619]	29.9 [14.3, 45.8]
Municipal C*: mixed surface (12-63%) & well	16.2%	949 [882, 882]	23.4 [13.1, 33.6]
Municipal D*: well, with added surface water in emergencies & summer	8.8%	1,252 [1150, 1150]	43.1 [24.1, 61.7]
Municipal E*: well, with added surface water in emergencies & summer	1.5%	829 [627, 627]	50.7 [1.0, 100]
Municipal F: well, large systems	14.9%	1,340 [1257, 1257]	54.4 [38.1, 70.6]
Municipal G: well, small systems	0.4%	412 [142, 142]	0 [0, 216]
Community well	1.7%	949 [747, 747]	11.3 [0, 33.5]
Private well	20.7%	949 [887, 887]	32.9 [22.1, 43.5]
Sewage Disposal			
Municipal sewer user	52.8%	1,701[1653, 1653]	39.4 [32.3, 46.9]
Private sewer user	47.3%	971 [930, 930]	27.0 [20.7, 33.6]
Land Use			
Agricultural	8.3%	1,059 [962, 962]	32.1 [15.2, 48.7]
Residential	91.7%	1,380 [1347, 1347]	33.9 [28.8, 39.1]

^a ^Number of physician visits = 7,017 in 189,234,765 person-days of observation

^b ^Number of hospitalizations = 180 in 194,552,383 person-days of observation

* = Systems receiving both well water from municipal wells in the Township and surface water from North Shore Mountain reservoirs. Areas A, B, and C receive mixed water throughout the year; the estimated proportions of surface water listed above are based on 2009 total, peak and minimum flows at central monitoring stations. Areas D and E receive surface water only in emergencies (e.g., system maintenance work) and in peak summer months when well water levels are low. Township personnel indicated that the water distribution network design suggests additional differences between areas that cannot be quantified, such that area A is believed to receive more surface water than area B, and area D is believed to receive more surface water than area E. The proportions of surface water in all five mixed systems vary with rainfall, system water use, and system maintenance.

Crude incidence rates of intestinal infectious diseases were 1,353 physician visits per 100,000 person-years (95% CI: 1,322-1,385/100,000) and 33.8 hospitalizations per 100,000 person-years (95% CI: 28.8-38.7/100,000). Table 2 lists the crude incidence rates for each category of each variable. These rates exclude potentially independent subsequent disease episodes after an initial episode. Among 7,017 subjects with a physician visit for an intestinal infectious disease, 23% (1,592/7,017) had multiple billing records for such diseases. Most (1,410/1,592) had the same ICD-9 codes for the initial and repeat visits, and more than half (835) had consecutive records less than 3 months apart making it very likely the records were for the same episode. Only 6.5% of subjects with a physician visit (454/7,017) had consecutive billing records more than one year apart. Among 180 subjects hospitalized for an intestinal infectious disease, 3.3% (6/180) had multiple hospital discharge records for such diseases, all with the same ICD-9 codes for the initial and repeat visits. Five of the 6 subjects with repeat records had second records less than a month after the initial hospitalization, and one had a second hospitalization more than a year later.

**Figure 2:**
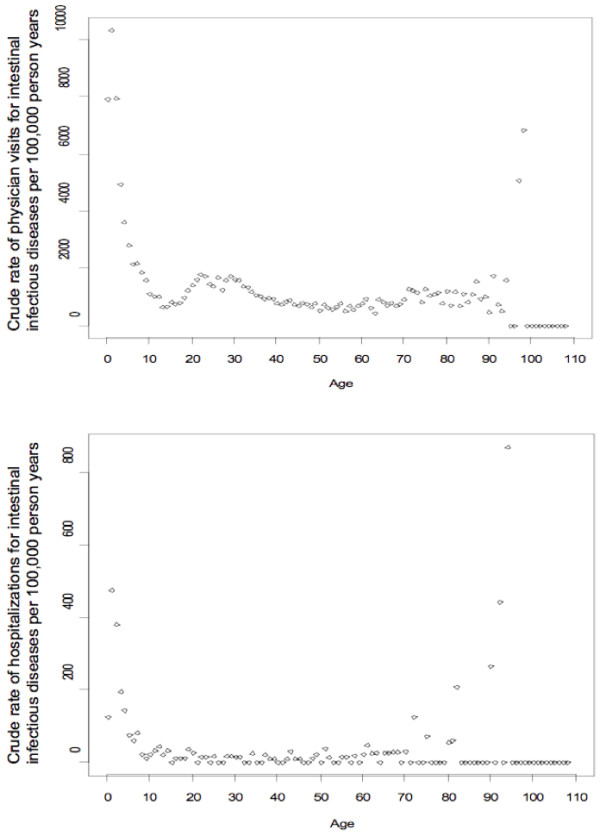
**Crude intestinal infectious disease incidence rates vs. age**. Data from 1995 to 2003 inclusive: a) N = 7,017 physician visits in 189,234,765 person-days of observation (top). b) N = 180 hospitalizations in 194,552,383 person-days (bottom).

Variability in crude incidence rates was greatest for age (Table 2 Figure 2). The pattern of higher rates at the extremes of age was similar for physician visits and hospitalizations The rates were consistently high among those under 5, whereas those in the oldest ages had more variable rates due to small numbers, especially among those over 90 years old for physician visits and over 70 years for hospitalizations (Figure 2). Physician visit rates were also higher among young adults (ages ~ 20-35), but this pattern was not observed for hospitalizations.

Patterns of incidence rates were similar for both physician visits and hospitalizations for many of the other independent variables (Table 2). Seasonal rates were highest in the spring and lowest in the fall. Those who had resided in the Township for fewer years had higher rates. Those living in neighborhoods with the two lowest household income quintiles had higher rates. Rates were lower with chlorinated water, with private sewage disposal systems, and agricultural land uses. There was no clear pattern related to water system, except that those with wells connected to a small number of properties and run either by a private community or by the municipality had the lowest rates.

Table 3 summarizes the results of logistic regressions examining associations between intestinal infectious diseases and the independent variables representing socio-demographic, temporal, and environmental factors. All variables, except land use, were significant in the analysis of physician visits, and supported the patterns observed for the crude incidence rates. The only variables with significant relationships to hospitalizations were age and season. Though not statistically significant, the patterns of the odds ratio estimates for most variables (except socioeconomic status, water system, and land use) were the same for hospitalizations and physician visits.

**Table 3:** Odds ratios^a^ and 95% confidence intervals for associations between physician visits or hospitalizations for intestinal infectious diseases and socio-demographic, temporal, and environmental variables.

	Physician Visits			Hospitalizations		
	
		Wald 95% Confidence Limits			Wald 95% Confidence Limits	
				
Variable & Category	Odds Ratio	Lower	Upper	Odds Ratio	Lower	Upper
Sex						
Female	**1.08**	1.03	1.13	1.18	0.88	1.58
Male	1.00	-	-	1.00	-	-
Age						
< 1	**5.22**	4.26	6.40	2.75	0.60	12.6
1 - 4	**4.83**	4.32	5.39	**6.54**	3.64	11.8
5 - 9	**1.81**	1.61	2.04	1.33	0.69	2.58
10 - 19	**0.80**	0.71	0.90	0.54	0.27	1.07
20 - 29	**1.31**	1.17	1.47	**0.26**	0.11	0.64
30 - 39	0.96	0.86	1.08	**0.14**	0.05	0.40
40 - 49	**0.69**	0.61	0.78	**0.22**	0.09	0.51
50 - 59	**0.63**	0.55	0.72	**0.20**	0.07	0.56
60 - 69	**0.69**	0.59	0.80	0.62	0.27	1.42
≥ 70	1.00	-	-	1.00	-	-
Season						
Spring	**1.08**	1.01	1.15	**1.45**	1.01	2.07
Summer	**0.89**	0.83	0.95	**0.55**	0.35	0.87
Fall	**0.77**	0.72	0.83	**0.50**	0.31	0.81
Winter	1.00	-	-	1.00	-	-
Duration of Residence in the Township						
< 1 year	**1.16**	1.03	1.30	1.04	0.46	2.35
1 - 2 years	0.98	0.88	1.09	0.89	0.40	1.95
2 - 3 years	**0.80**	0.70	0.91	0.73	0.30	1.78
3 - 6 years	**0.73**	0.64	0.82	0.62	0.26	1.49
≥ 6 years	**0.69**	0.60	0.80	0.55	0.20	1.57
Unknown	1.00	-	-	1.00	-	-
Household income quintile of the neighborhood						
Low	**1.18**	1.03	1.34	1.17	0.58	2.37
Medium Low	**1.19**	1.07	1.32	0.91	0.49	1.69
Medium	1.06	0.98	1.15	0.85	0.51	1.42
Medium High	1.02	0.95	1.09	0.89	0.57	1.39
High	1.00	-	-	1.00	-	-
Drinking Water Disinfection						
Chlorination	**0.92**	0.85	1.00	0.89	0.53	1.48
None	1.00	-	-	1.00	-	-
Water System or Subsystem						
Municipal A: mixed surface (66-96%) & well	**1.57**	1.39	1.78	0.54	0.24	1.20
Municipal B: mixed surface (66-96%) & well	**1.45**	1.28	1.64	0.66	0.29	1.52
Municipal C: mixed surface (12-63%) & well	1.01	0.90	1.12	0.71	0.36	1.40
Municipal D: well, with surface water added in emergencies & summer	1.00	0.87	1.15	0.83	0.37	1.87
Municipal E: well, with surface water added in emergencies & summer	0.89	0.69	1.15	1.35	0.45	4.03
Municipal F: well, large systems	0.96	0.85	1.08	0.93	0.45	1.94
Municipal G: well, small systems	**0.51**	0.27	0.99	0	0	> 999
Community well	1.04	0.83	1.30	0.34	0.05	2.49
Private well	1.00	-	-	1.00	-	-
Sewage Disposal						
Municipal sewer	**1.26**	1.14	1.38	1.42	0.77	2.62
Private sewage system	1.00	-	-	1.00	-	-
Land Use						
Agricultural	1.06	0.95	1.19	0.95	0.51	1.80
Residential	1.00	-	-	1.00	-	-

^a ^Based on unconditional logistic regression, also adjusted for calendar year. Statistically significant odds ratios in bold.

- Reference value, no confidence limits calculated.

Well depth was known for 3,815 wells. The mean depth was 36.6 m, with a recorded range of 0 to 280 m. No relationship between well depth and physician visits for intestinal infectious diseases was observed in crude incidence rates or in logistic regression modelling restricted to those living at properties whose well depth was known. Crude incidence rates (and proportion of total person-time) in three well depth categories were: 1,049/100,000 person-years (2.0% of person-time) for well depths up to 9 m; 1,089 (6.2%) for depths of greater than 9 to 30 m; and 1,032 (8.2%) for depths greater than 30 m.

**Figure 3:**
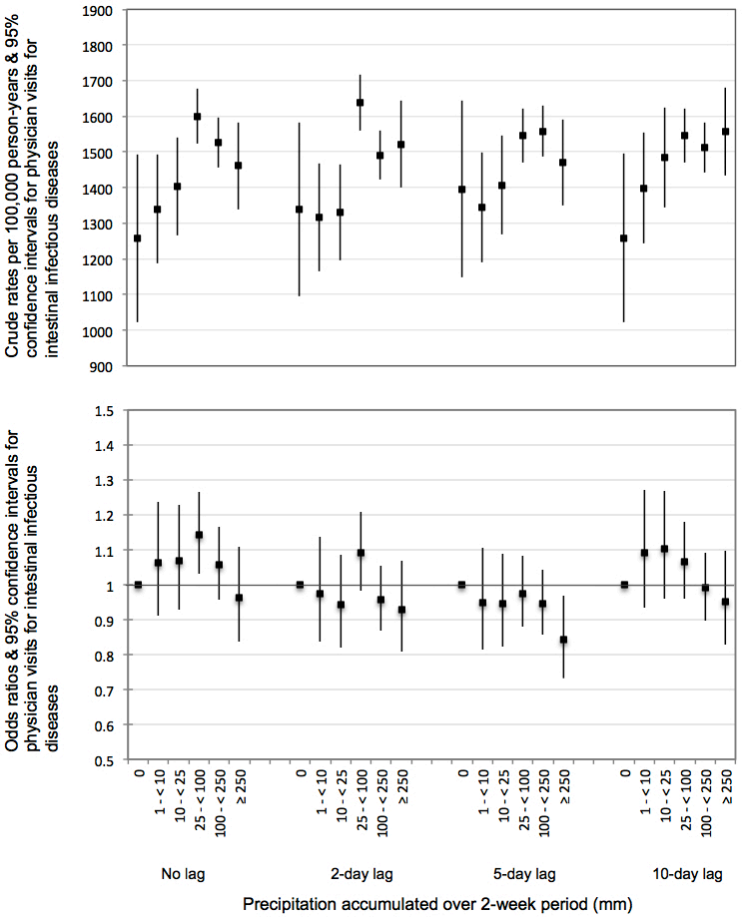
**Physician visits for intestinal infectious diseases vs. precipitation accumulated over prior 2 weeks**. Data from 1995 to 2003 inclusive; restricted to subpopulation (N = 4,868 physician visits and 117,543,309 person-days of observation) that received unfiltered surface water from North Shore Mountain reservoirs. a) Crude incidence rates per 100,000 person-years (top). b) Odds ratios adjusted for sex, age, calendar year, season, duration of residence in the Township, neighborhood household income quintile, drinking water disinfection, water system, sewage disposal, and land use (bottom)

The association between precipitation and physician visits for an intestinal infectious disease was examined for those residents receiving municipal water from the North Shore mountain surface water reservoirs (62.1% of person-time). No relationship was observed for one-week rainfall accumulations. For two-week accumulations, crude rates suggested a pattern of increasing illness with increasing rainfall, particularly with a 10-day lag (Figure 3a). However, in logistic regressions adjusting for all other variables, the trends did not hold (Figure 3b).

The response rate for the survey of Township households was 59% (N = 546 respondents of 926 eligible; N = 74 were ineligible, including businesses, vacant addresses, mail returned to sender, etc.). Of those responding, 62% received municipal water, 5% community water, and 33% private well water. Almost all households used tap water for cooking (92%, 95% CI: 90-94%), most also used it for drinking (79%, CI: 76-82%), and a large proportion filtered their tap water (45%, CI: 41-49%). These proportions did not vary by water source. Most respondents with private wells tested their water for coliform bacteria (71%, CI: 67-75%) and for nitrates (68%, CI: 64-72%). Almost all (88%, CI: 85-91%) knew their well depth, with reported depths ranging from 3 to 152 m and a mean of 39 m, very similar to the mean for wells in the Ministry of Environment database. About 35% (CI: 31-39%) of respondents with private wells reported problems with their water. Most were related to minerals and sediment (reported for 17% of all wells) or the well running dry (5%), but 12% reported bacteriological contamination at some point. Contaminated wells were treated, about half with bleach and the other half by installing ultraviolet light disinfection systems. Of those with private sewage systems (49%; CI: 45-53%), 97% reported that the system was a septic tank and drain field. The few other types included engineered systems with biofilters or small treatment plants.

## Discussion

### Disease rates

The crude incidence rates of intestinal infectious diseases found in this study (1,353 physician visits and 33.8 hospitalizations per 100,000 person-years) are difficult to compare to those reported elsewhere, because of different case definitions and identification methods. The most similar data are for enteric disease hospitalizations in the US from 1998 to 2006; Christensen *et al. *[20] found a rate of 56.6/100,000, somewhat higher than in this study, likely because they included more ICD-9 codes (001-009, 022.2). Tinker *et al. *[13] reported emergency department visits for gastrointestinal illnesses in Atlanta and found a rate of about 800/100,000. In a summary of several of his Canadian and US studies, Payment [21] estimated a self-reported gastrointestinal illness (nausea, vomiting, diarrhea) rate of 70,000/100,000. In a modeling exercise to estimate the rate of acute gastrointestinal illness specifically due to drinking water in the US, Messner *et al. *[22] estimated a rate of 6,000/100,000.

### Environmental factors

The main focus of this study was whether environmental variables related to water quality were associated with intestinal infectious diseases. Chlorination was associated with lower physician visit and hospitalization rates (latter not statistically significant). Even the small reduction in risk suggested by these data may have public health importance, since this is one of the dominant methods of water treatment. Although chlorination has been shown to be effective in preventing enteric disease outbreaks [23], the few reports investigating endemic disease have been less clear. Hellard *et al. *[11] examined a children's hospital emergency visits (numerator data only) before and after chlorination of the Melbourne, Australia water supply, but found no change. Odoi *et al. *[15] found no difference in giardiasis rates between areas in Ontario, Canada with and without chlorination.

This study was able to examine numerous types of water systems, and to examine sub-classifications of those systems. We expected that those with private well water would have higher enteric disease rates than those with municipal and community well water, as found by others [16,24-26], though not all [17]. Beyond the effect of chlorination, this was not the case. Two small municipal well water supplies each serving fewer than 100 homes had physician visit rates that were about half those of the private wells, but other municipal and community well water sources had rates similar to private wells. Well depth was not associated with differences in endemic disease rates, lending support to lack of influence of private well water on disease rates, though it is important to note that the subset of wells with known depth was likely to have been correctly constructed to prevent contamination, since well depths were voluntarily reported by professional well drillers. Evidence from the survey suggests that most Township residents with private wells were well informed about their water systems and conscientious about testing them and correcting problems.

We also expected that those served by municipal surface water supplies would have higher disease rates than those served exclusively by municipal well water systems, as found elsewhere [15,17,24,26,27]. There was some evidence of this in the physician visit but not the hospitalization data. Of the 5 systems that had mixed surface and well water, the two with the highest proportions of surface water had the highest physician visit rates, about 50% higher than those served by most other systems types, including those with smaller proportions of surface water. We further investigated the systems receiving surface water, to examine the impact of recent precipitation. While crude rates suggested increased physician visits with increased precipitation, this trend was not robust to adjustment for other variables. A number of investigators have examined both precipitation and the turbidity it can induce. Curriero *et al. *[28] found that more than half of surface water-related outbreaks were preceded by extreme precipitation in the month prior. Aramini *et al. *[10] studied Vancouver, which receives all its drinking water from the same protected North Shore mountain watersheds that partially serve areas of the Township. They found associations between increased turbidity and endemic gastroenteritis, as measured by physician visits and hospitalizations. Others have observed similar relationships [8,14,29]. In all studies, the associations were small, but significant. These studies did not include the range of variables available about the Township and its residents, so it is unknown whether controlling for other factors would have diminished the associations, as occurred in our analyses.

Although the relationship between sewage contamination and enteric disease outbreaks is well established [30], we identified only two other studies that examined endemic disease risk by sewage system type. Denno *et al. *[16] found higher odds ratios for *Salmonella *infection for those with private septic systems. Febriani *et al. *[17] found no difference in gastrointestinal illness prevalence between those with private and municipal sewage disposal. We expected private septic systems might have drain-field-to-well contamination that could result in higher disease rates. Instead higher disease rates were observed among those with municipal sewer connections for both physician visits and hospitalizations (the latter not statistically significant). This result was apparent in the initial crude analyses and remained after adjustment for all other factors in the models, including the neighborhood-level adjustment allowed by the water system variable. To check whether municipal sewer might be a surrogate for other factors not considered *a priori*, we conducted *post hoc *analyses including population density and distance to nearest hospital. Neither variable was associated with disease events and the higher odds ratios for municipal sewer remained stable. We also examined disease rates for the interaction of sewage and water system types. There was no difference in rates between private and municipal sewage systems for those with private well water, but those with both municipal sewer and municipal water systems, whose pipes are likely to run along similar paths, had higher disease rates. Although the higher disease rates for municipal sewer users may be a chance finding or may be related to other factors not accounted for in this study, it is prudent to consider possible explanations. The Township does not have combined storm and sanitary sewer lines, so overflow is not an issue. Water lines are required to be separated from sewer lines by 0.5 m vertically (sewer lines below) and 3 m horizontally, and where these conditions cannot be met, the water main is to be protected from infiltration by wrapping the pipe joints with petrolatum tape. Although there is no comparable data from the Township, some studies have shown possible modes of contamination despite the care taken when pipes are laid. A US study described sources of sewer line leakage and concluded exfiltration can occur where the groundwater table is below the sewer lines (as is the case in the Township) [31]. Others have found that soil and water samples next to water lines frequently have fecal contamination [32], and that breaks and maintenance work on water lines [12,23] and low water pressure [33] are related to gastrointestinal illness.

### Sociodemographic and temporal factors

Sociodemographic and temporal factors were also associated with infectious intestinal disease rates in this study. Females had higher rates than males in our study, as found elsewhere [20]. The age distributions of physician visits and hospitalizations largely followed a u-shaped pattern of high rates in the very young and elderly, and for physician visits only, of increased rates in young adults as well (an association often thought to be related to travel or parenting young children). Christensen *et al. *[20] reported the age distribution of hospitalizations for all infections (not just enteric), and the u-shaped pattern was similar to that observed here. The demographic differences in disease rates are likely, in part, to reflect differences in health care utilization across these characteristics.

Hospitalizations and physician visits for enteric diseases were highest from December through May and lowest from June through November, the same pattern observed in a Quebec study of gastrointestinal illness prevalence [17]. In a study of physician billings for enteric diseases in the neighboring Canadian province of Alberta, the highest rates were observed from November through March, and the lowest in September [34]. Christensen *et al. *[20] examined seasonality for all infectious diseases in the US and found the highest incidence from December through March inclusive and the lowest incidence from June to September. Slight offsets in seasonality may in part reflect differences in climates between the jurisdictions studied.

Lower household income (as an ecological variable, for each census dissemination area) was related to increased physician visits for infectious intestinal diseases, not surprising since income has been similarly associated with many health indicators in Canada, including life expectancy [35]. Studies specific to enteric disease have also found increased rates for those with lower income [15]. A study by Tam et al. [36] raises the question of the extent to which these results reflect differences in disease rates or differences in likelihood of attending a physician by socio-economic status.

Finally, duration of residence was consistently related to disease rates. Those who lived in the Township longer had lower rates of both physician visits and hospitalizations (the latter not statistically significant). Febriani *et al. *[17] identified a similar pattern. Frost *et al. *[37] showed that episodes of gastrointestinal illnesses were 35 to 60% lower among subjects with antigens to *Cryptosporidium parvum*, suggesting that an explanation of this finding may be increasing immunity to local pathogens over time. It may also be possible that individuals are likely to seek medical advice for enteric illnesses when they first move to an area, but may be less likely to do once they are established in the community.

### Strengths and limitations

This study had the following advantages: a large cohort with almost a decade of follow-up; a cohort design which allowed calculation of disease incidence rates; objective data on dependent and independent variables of interest; and survey data about water-related behaviors in the study area.

There were also limitations. The administrative data on disease events were provided by physicians, not laboratories, therefore clinical suspicion was used to assign general rather than specific disease coding, preventing analyses of individual diseases such as giardiasis or cryptosporidiosis. Because it is difficult to specify a between-event period to define second incident events within an individual, we included only first events. The incidence rates for physician visits and hospitalizations are therefore underestimates, though the distribution of subsequent events suggests that this is a small problem, representing an underestimate of 10% or less. Intestinal infectious diseases do not always result in contacts with the health care system, so the incidence rates calculated here omit less severe events and events among people less inclined to visit a physician [36]. If the propensity to visit a physician is related to the main environmental variables of interest, the effect estimates could be biased. We did *post-hoc *analyses to adjust for factors thought to be related to the propensity for physician visits and hospitalizations (distance to the nearest hospital, population density) and these did not change the results for the environmental variables. In fact, the physician visit results for all independent variables, except land use and precipitation, were stable from the crude rates to the adjusted analyses.

Many of the independent variables, including environmental variables, household income quintile, and duration of residence in the Township, were dependent on the temporal and spatial accuracy of residential address records held by the Client Registry of the Ministry of Health Services. Since address records are updated when the health care system is used, the timeliness of address changes may be related to overall health status. We tested the reliability of selected administrative data via comparisons to data collected in the survey. Despite the 3-year offset between these two data sources and potential errors in the survey data, kappas for agreement beyond chance were 0.76 for water system type and 0.78 for sewage system type, indicating very good reliability. Finally, details on certain features were not available, for example well and septic system ages, and water quality at the tap of individual residences.

## Conclusions

The study is one of few that have examined risk of physician visits and hospitalizations for endemic infectious intestinal diseases across an array of water and sewer system types. Chlorination of water supplies was shown to be associated with lower risks. Surface water was associated with higher risks in two of five systems. Private well water was not associated with increased risk, likely because of awareness of water quality and quantity issues by Township residents. Those with municipal sewer systems appeared to have increased disease risk, though this result needs to be viewed with caution since it was not anticipated and has not been observed elsewhere. Further studies to examine disease incidence among populations with municipal versus private sewage systems are warranted. Most socio-demographic variables had predicted associations, with higher physician visit and hospitalization rates in females, in the very young and very old, and in those in low income areas. Increased duration of residence in the Township was associated with reduced risk, perhaps due to increasing immunity to local pathogens over time.

For public health and civil engineering personnel, these results reinforce confidence in chlorination as a means of reducing enteric disease risk, and indicate that private well water users who understand their systems and know how to respond to water quality issues identified during monitoring can minimize disease risks. The results also convey cautions: they support previous research indicating that surface water sources, even with protected watersheds, deserve special attention; and present new data suggesting municipal sewer systems may require more scrutiny than previously thought.

The results of this study, which did not always follow prior expectations, underscore the importance of studying factors associated with endemic disease across water and sewage system types.

## List of Abbreviations

BC: Canadian province of British Columbia; CI: confidence interval; ICD-9: International Classification of Diseases, 9^th ^edition; OR: odds ratio

## Competing interests

The authors declare that they have no competing interests.

## Authors' contributions

KT led the design, conduct, and analysis of the study and drafted the paper. NB led the environmental data gathering, conducted the ArcGIS linkage, liaised with the Ministry of Health Services for the linkage of the environmental data to the cohort data, and participated in interpretation of results and manuscript review. H Shen conducted the data analyses and participated in interpretation of results and various stages of the manuscript reviews and revisions. JA had the initial idea for the study, and participated in the design, analyses, interpretation, and manuscript revisions. RC managed the linked dataset, screened the cohort for eligibility, and used Ministry billings data to define cases. MK led the application to Population Data BC, participated in data analyses, interpretation, and manuscript review. YCM participated in the study design, analyses, interpretation and in various stages of the manuscript reviews and revisions. H Schreier, and JLI-R participated in the study design, analyses, interpretation and manuscript review. All authors have read and approved the final manuscript.

## Pre-publication history

The pre-publication history for this paper can be accessed here:

http://www.biomedcentral.com/1471-2458/10/767/prepub
